# Supplementary material for: MetaRibo-Seq measures translation in microbiomes
Source: Nat Commun. 2020 Jun 29;11:3268. doi: 10.1038/s41467-020-17081-z (PMC7324362; doi:10.1038/s41467-020-17081-z)
Supplement: Supplementary file 10 — Supplementary Data 7 [file 41467_2020_17081_MOESM10_ESM.zip › File2/Confidence_High_Taxonomy/109255_out.krona.html]

Javascript must be enabled to view this page.

members
magnitude
magnitudeUnassigned
count
unassigned
taxon
rank

109255\_out

54

54
2
superkingdom

phylum
976
54

54
class
200643

171549
order
54

1853231
family
1

1
283168
genus

1
28118

SRS143598\_contig\_number\_11980
species

815
family
53

genus
816
16
53

SRS013476\_contig\_number\_12541SRS013800\_contig\_number\_10658SRS013951\_contig\_number\_16635SRS015794\_contig\_number\_229SRS015890\_contig\_number\_2728SRS075963\_contig\_number\_312SRS098655\_contig\_number\_293SRS1041136\_contig\_number\_contig-100\_47.75891SRS1054691\_contig\_number\_12538SRS1055067\_contig\_number\_1821SRS147139\_contig\_number\_23880SRS148874\_contig\_number\_898SRS149879\_contig\_number\_5318SRS893230\_contig\_number\_8689SRS893300\_contig\_number\_9745SRS971276\_contig\_number\_9328

2
28116

SRS013215\_contig\_number\_192SRS1041112\_contig\_number\_843
species

1
2292944

SRS043768\_contig\_number\_3160
species

1263042

SRS043001\_contig\_number\_320SRS056519\_contig\_number\_422SRS098717\_contig\_number\_195
species
3

1
469593
species

SRS078419\_contig\_number\_399

1
species

SRS077024\_contig\_number\_3708
47678

1

SRS046717\_contig\_number\_931
species
291645

20
357276

SRS013940\_contig\_number\_7040SRS016018\_contig\_number\_409SRS018049\_contig\_number\_162SRS018049\_contig\_number\_255SRS018133\_contig\_number\_906SRS018541\_contig\_number\_196SRS018984\_contig\_number\_1928SRS019286\_contig\_number\_19SRS024549\_contig\_number\_528SRS024663\_contig\_number\_2597SRS045195\_contig\_number\_1241SRS048981\_contig\_number\_722SRS052078\_contig\_number\_1098SRS054590\_contig\_number\_667SRS075021\_contig\_number\_699SRS075341\_contig\_number\_7116SRS101268\_contig\_number\_26SRS1055034\_contig\_number\_419SRS140645\_contig\_number\_13SRS142712\_contig\_number\_1307
species

818

SRS023346\_contig\_number\_630SRS057717\_contig\_number\_2294
species
2

species

SRS016381\_contig\_number\_2540SRS149784\_contig\_number\_18017
671267
2

species

SRS077127\_contig\_number\_136
93975
1

821

SRS1041038\_contig\_number\_9417
species
1

2
species

SRS048060\_contig\_number\_4079SRS1041145\_contig\_number\_10499
2292935
